# Phonological-Lexical Feedback during Early Abstract Encoding: The Case of Deaf Readers

**DOI:** 10.1371/journal.pone.0146265

**Published:** 2016-01-05

**Authors:** Manuel Perea, Ana Marcet, Marta Vergara-Martínez

**Affiliations:** Universitat de València, Valencia, Spain; The National Institutes of Health, UNITED STATES

## Abstract

In the masked priming technique, physical identity between prime and target enjoys an advantage over nominal identity in nonwords (GEDA-GEDA faster than geda-GEDA). However, nominal identity overrides physical identity in words (e.g., REAL-REAL similar to real-REAL). Here we tested whether the lack of an advantage of the physical identity condition for words was due to top-down feedback from phonological-lexical information. We examined this issue with deaf readers, as their phonological representations are not as fully developed as in hearing readers. Results revealed that physical identity enjoyed a processing advantage over nominal identity not only in nonwords but also in words (GEDA-GEDA faster than geda-GEDA; REAL-REAL faster than real-REAL). This suggests the existence of fundamental differences in the early stages of visual word recognition of hearing and deaf readers, possibly related to the amount of feedback from higher levels of information.

## Introduction

Current neural models of visual word recognition assume that, for skilled hearing readers, there is rapid access to orthographic, phonological, and lexical representations (e.g., crash and CRASH would activate the same codes; see [1–3]). This assumption is consistent with evidence from Forster and Davis’ [4] masked priming technique (i.e., a paradigm that taps the early stages of word processing; for review, see [5, 6]). An excellent illustration is the masked priming lexical decision experiment reported by Jacobs, Grainger, and Ferrand [7]. Jacobs et al. [7] found that, for word targets, briefly presented (and forwardly masked) matched-case identity primes were as effective as mismatched-case identity primes (e.g., YEUX-YEUX and yeux-YEUX [the French for eyes] produced similar response times). That is, the physical differences between prime and target in pairs such as yeux-YEUX were entirely overridden by nominal (abstract) similarity (see also [8–10] for converging evidence in English and Spanish). This phenomenon occurs not only with high-frequency words, but also with low-frequency words (e.g., see [8]). Furthermore, Perea et al. [8, 9] found that, for adult readers and for developing readers (Grade 5 children), this pattern occurs for cross-case physically similar words (e.g., SIM words; KISS-KISS similar to kiss-KISS) and cross-case physically dissimilar words (e.g., DIS words; REAL-REAL similar to real-REAL). This finding is important to discard the possibility that these cross-case effects originated at some level of the visual representation. To examine in detail the time course of this phenomenon, Vergara-Martínez et al. [10] recorded event-related potentials (ERPs) in a masked priming lexical decision experiment. They found that the ERP waves associated to orthographic-phonological processing and to lexical-semantic processing (N250 and N400, respectively) were virtually the same for word targets preceded by matched- and mismatched-case identity primes. Indeed, Vergara-Martínez et al. [10] only found differences between the ERP waves of these two conditions in an early component associated to the processing of visual features (N/P150) (see also [11, 12] for parallel evidence using fMRI).

There is some evidence—in a non-alphabetic writing system (Japanese)—that suggests the existence of common phonological-lexical codes may be sufficient to override the influence of physical similarity. Besides Kanji (i.e., ideograms taken originally from Chinese), Japanese words can be written in one of two syllabaries (Hiragana and Katakana). Each grapheme in Katakana and Hiragana represents a syllabic sound. For instance, the Hiragana character ぴ and the Katakana character ピare associated to the same sound, the syllable /pe/, but they do not share the orthography. As Okano, Grainger, and Holcomb [13] indicated, there would presumably be “greater use of phonological information when processing words written in a syllabary script, given that these scripts were specifically designed to facilitate such phonological processing.” (p. 401) Although Katakana and Hiragana words are used in different contexts (e.g., Katakana is used for loan words or foreign names/places), any Japanese word can be written in Katakana or Hiragana (e.g., pepper would beぴいまんin Hiragana andピーマン in Katakana). In a masked priming lexical decision experiment, Pylkkanën and Okano [14] found that the response times to target words in Katakana were similar when preceded by an identity prime in Hiragana (e.g., ぴいまん-ピーマン) and when preceded by an identity prime in Katakana (e.g., ピーマン-ピーマン). Note that, unsurprisingly, these two conditions produced faster response times than the unrelated priming condition. Given that Katakana and Hiragana words only share the phonology (i.e., sound identity), the Pylkkanën and Okano [14] findings suggest that the presence of common phonological codes, as in ぴいまん-ピーマン, is sufficient to override the physical differences in the early stages of word recognition.

The similar response times and ERP correlates for word targets preceded by a matched- vs. mismatched-case identity prime—or a within-script vs. cross-script identity prime—are consistent with the assumption of an early prelexical stage in which the retinotopic representations of the word’s constituent letters are transformed onto shared orthographic and phonological codes. However, there is a phenomenon that rules out a purely bottom-up interpretation of this effect. A number of masked priming experiments in different languages (French, English, Spanish) have consistently found that, for nonword targets, matched-case identity primes are *more effective* than mismatched-case identity primes (GEDA-GEDA faster than geda-GEDA; see [7, 8, 10]). If the orthographic and phonological codes during the process of visual word recognition were initially formed in a purely bottom-up manner, one would have expected similar response times (and ERP waves) for nonword targets when preceded by a matched-case identity prime and when preceded by a mismatched-case identity prime. Furthermore, the matched-case advantage for identity primes occurs to the same degree with cross-case visually dissimilar pairs (DIS nonwords; GEDA-GEDA faster than geda-GEDA) and cross-case visually similar pairs (SIM nonwords: CIKY-CIKY faster than ciky-CIKY) (see [8]). When examining the electrophysiological correlates of this phenomenon, Vergara-Martínez et al. [10] found that the ERP waves in the matched- and mismatched-case identity priming conditions with nonword targets differed not only in an early visual component (N/P150), but also in later components (N250 and N400). Vergara-Martínez et al. [10] indicated: “This divergence occurs because there is no feedback from higher-level processes, because pseudowords do not have lexical representations.” (p. 500)

A parsimonious explanation of the word/nonword dissociation in masked identity priming is that, unlike nonwords, words benefit from top-down feedback from the phonological and lexical-semantic levels in a fully interactive model of visual word recognition ([10]; see Figure 2 in [1] for illustration). Indeed, in their neural model of visual word recognition, Dehaene et al. [2] claimed: “feedback and lateral connections are numerous in the visual system, and probably contribute to shaping the neurons’ receptive field.” (p. 338) This top-down feedback would help achieve stable phonological codes for words like real and REAL in the early stages of processing, thus leading to similar neural correlates for REAL-REAL and real-REAL in masked priming experiments. In contrast, as nonwords do not have stored representations in the lexicon, they would not benefit from top-down feedback. As a result, matched-case identity pairs (GEDA-GEDA) would enjoy a processing advantage over mismatched-case identity pairs (geda-GEDA).

While the Vergara-Martínez et al. [10] findings are entirely consistent with the claims of fully interactive activation models [1], a remaining question is which pathway is responsible for the lack of a visual effect with word stimuli. In the present experiment, we examined whether the activation of common phonological codes may be responsible for the lack of differences between REAL-REAL and real-REAL. In particular, we examined the hypothesis that the lack of an advantage of physical+nominal vs. nominal pairs in masked identity priming is due to top-down feedback from shared phonological-lexical information. To that end, we conducted a masked priming lexical decision experiment with deaf readers. The rationale is that phonological representations in deaf readers are not as fully and consistently developed as the phonological representations in hearing readers. This is because phonological codes in deaf readers do not primarily originate from an auditory channel, but rather from speech production or visual lip reading (e.g., see [15, 16]).

Previous masked priming experiments with deaf readers failed to find evidence of activation of phonological codes in the early stages of word processing [15, 17]. Cripps et al. [17] found a facilitative masked phonological priming effect in hearing readers (bloo-BLUE faster than caft-BLUE; 22 ms), but not in deaf readers. More recently, Bélanger et al. [15] examined masked orthographic priming (e.g., keit–KAÎT vs. kets–KAÎT) and masked phonological priming (e.g., kets-KAÎT vs. kaum–KAÎT) in a lexical decision experiment with hearing readers and skilled and less skilled deaf readers. For hearing readers, Bélanger et al. [15] found significant masked orthographic and phonological priming effects. For deaf readers, Bélanger et al. [15] found significant masked orthographic priming effects, but not phonological priming. Importantly, the lack of masked phonological priming occurred both in skilled and less skilled deaf readers. Similar evidence was obtained with another technique that taps early processes during sentence reading, Rayner’s [18] gaze contingent boundary change paradigm. Specifically, Bélanger, Mayberry, and Rayner [19] conducted a gaze contingent boundary change experiment, in which the prime was presented as a parafoveal preview and replaced by the target upon fixation. While skilled hearing and deaf readers (both skilled and less-skilled) showed an orthographic preview benefit, only skilled hearing readers showed a phonological preview benefit. Taken together, these findings suggest that phonological recoding “is an automatic process at some stage of visual word recognition in hearing people but not for deaf people” ([17], p. 41). In this line, recent fMRI evidence has revealed a similar pattern of activation in the so-called visual word form area (VWFA) in both hearing and congenitally deaf readers, yet its functional connectivity strength with speech-related regions in the left anterior superior temporal gyrus was reduced in the deaf group [20]. We acknowledge, however, that deaf readers may develop stable phonological representations with some training [21]. Nevertheless, this phonological activation may occur at a post-lexical level rather than at the earliest stages of word processing—as deduced from the lack of masked phonological priming even in skilled deaf readers.

Thus, deaf readers may not benefit from the activation of phonological codes in the early stages of word processing. As phonological-lexical information may help stabilize the initial formation of shared codes for pairs such as real-REAL (or kiss-KISS) [13], matched-case identity pairs in deaf readers may enjoy a processing advantage over mismatched-case identity pairs not only in nonwords (e.g., GEDA-GEDA faster than geda-GEDA), but also in words (e.g., REAL-REAL faster than real-REAL). Note that the creation of shared abstract units for lowercase and uppercase letters may be mediated by the activation of common phonological codes (e.g., “a” and “A” would activate the same sounds; see [22]), a process that may be hindered in deaf readers (but see Polk et al. [23] for an alternative account that does not require phonological codes). In the present masked priming lexical decision experiment, a target stimulus—always in uppercase—could be preceded by: i) a matched-case identity prime; or ii) a mismatched-case identity prime. To avoid visual continuity between prime and target, we added a 16.6-ms pattern mask between the prime and the target (i.e., 500-ms pattern mask + 33.3-ms prime + 16.6-ms pattern mask + target, so that the stimulus-onset asynchrony was 50 ms; see [7–10] for a similar procedure). For comparison purposes with previous research, we also included a third priming condition: an unrelated prime. As Forster [5] noted, the magnitude of masked repetition priming relative to the unrelated condition is sizable for word targets, whereas it is substantially smaller (and sometimes unreliable) for nonword targets (see also [24, 25]).

In the present experiment, we also examined if cross-case visual similarity between prime and target modulates the magnitude of masked priming effects. Half of the words (nonwords) were composed of letters that look visually similar in lowercase and uppercase (SIM words like kiss [KISS]), whereas the other half of the words (nonwords) was composed of letters that look visually dissimilar in lowercase and uppercase (DIS words like real [REAL]). Previous research with adult hearing readers failed to show any differences in masked priming effects for SIM vs. DIS words: 1) the magnitude of masked identity priming relative to an unrelated priming condition was similar for SIM and DIS words (e.g., soon-KISS minus kiss-KISS similar to able-REAL minus real-REAL; see [8, 9, 26]); and 2) matched-case and mismatched-case identity primes behave similarly for SIM and DIS stimuli (words: KISS-KISS similar to kiss-KISS; REAL-REAL similar to real-REAL; nonwords: GEDA-GEDA faster than geda-GEDA; CIKY-CIKY faster than ciky-CIKY; see [8, 9]). Nonetheless, one might argue that the lack of activation of phonological codes in the early stages of word processing in deaf readers may lead to increased sensitivity to the visual features of the letters. If this were the case, we would expect larger matched-case identity priming effect for the DIS compared to the SIM stimuli (the visual disruption between low-case prime and upper-case target is larger for DIS stimuli than for SIM stimuli). Because of the limited number of SIM and DIS words, each target word/nonword was presented three times, one in each priming condition (i.e., matched-case identity; mismatched-case identity; unrelated; see [8, 9, 10] for a similar procedure).

In summary, if phonological-lexical codes work as an early stable interface for pairs such as real-REAL (or kiss-KISS or ぴいまん-ピーマン) (see [14]), matched-case identity pairs in deaf readers may enjoy a processing advantage over mismatched-case identity pairs not only for nonwords (e.g., GEDA-GEDA faster than geda-GEDA), but also for words (e.g., REAL-REAL faster than real-REAL). Unlike their hearing counterparts, deaf readers may not benefit from the activation of phonological codes in the early stages of word processing. As the lack of activation of phonological codes in deaf readers may potentially lead to increased sensitivity to the visual features of the letters, we also examined if visual similarity between prime and target modulates the magnitude of masked priming effects.

## Materials and Methods

### Participants

Thirty-two deaf individuals from Valencia’s Deaf Community in Spain took part voluntarily in the experiment (M_age_ = 36; range: 18–61; 11 female). The Experimental Research Ethics Committee of the Universitat de València (Spain) specifically approved this study. Written consent was obtained from all the participants. Twenty-nine of them reported having profound hearing loss (> 90 dB in the better ear) and three reported having severe hearing loss (> 71 dB in the better ear)—two participants had a cochlear implant and were excluded from the final analysis. All participants were congenitally deaf or had their hearing loss in the first months of life. Eight participants had deaf, signing parents. Their usual mode of communication was Spanish Sign Language. All the participants had learned Spanish Sign Language as their first language before the age of 6. All participants had been educated in schools in Spanish, either in schools for the deaf or in integration schools. Their average self-assessed speech articulation ability was 6.1 in a 0–10 scale. All but three participants had finished at least secondary school Three of the participants did not perform the lexical decision task correctly (error rates above 30%) and were excluded from the analyses (i.e., the final sample was composed of twenty-seven participants).

### Materials

We selected a set of forty-eight Spanish words that were spelled with four letters. Twenty-four of these words were composed of (three/four) letters that were visually dissimilar across case (e.g., real-REAL) (mean word frequency per million in the Davis & Perea [27] B-Pal database: 53.6; range: 2.1–531.8; mean Coltheart’s N: 9.8; range: 0–25). The remaining twenty-four of these words were composed of (three/four) letters that were visually similar across case (e.g., vivo-VIVO [the Spanish for alive]) (mean word frequency per million: 56.7; range: 0.5–598.2; Coltheart’s N: 5.6; range: 0–15) (all *p*s > .25). In addition, the mean log (token) bigram frequencies were 1.43 for the SIM words (range: 0.25–2.71) and 1.59 for the DIS words (range: 0.32–2.94) (*p* > .40). As in previous research (see [8, 9]), the similar/dissimilar cross-case letters were selected on the basis of the Boles and Clifford [28] ratings (the cross-case visually dissimilar letters were: a/A, b/B, d/D, e/E, l/L, g/G, h/H, and r/R; the cross-case visually similar letters were: c/C, i/I, k/K, m/M, n/N, s/S, t/T, u/U, v/V, and w/W). We also created two sets of twenty-four pronounceable nonwords with the same characteristics as the words (i.e., DIS nonwords like GEDA, and SIM nonwords like BITU). The mean Coltheart’s N were 1.1 (range: 0–8) for the SIM nonwords and 2.4 (range: 0–14) for the DIS nonwords (*p* > .10). In addition, the mean log (token) bigram frequencies were 2.37 for the SIM words (range: 0.4–3.22) and 2.41 for the DIS words (range: 1.23–3.3) (*p* > .28). All the words in the experiment had a corresponding sign in Spanish sign language. The complete list of stimuli is presented in the Appendix. The target was always presented in uppercase and it was preceded by a prime stimulus that was: i) the same as the target, also in uppercase (e.g., REAL-REAL; VIVO-VIVO); ii) the same as the target, except that it was presented in lowercase (e.g., real-REAL; vivo-VIVO); iii) an unrelated prime stimulus (half in lowercase, half in uppercase)—we employed an unrelated word for word targets and an unrelated nonword for nonword targets. Each target stimulus was presented in each of the three conditions, thus leading to 24 trials per condition. As usual in masked priming experiments, the stimuli were presented with a monospaced font (14-pt Courier New).

### Procedure

Participants were tested individually. We employed a Windows OS computer equipped with DMDX software to present the stimuli and collect the responses [29]. In each trial, a forward pattern mask (####) was presented for 500 ms. Then, a prime stimulus was presented for 33.3 ms (i.e., two refresh cycles in the 60-Hz CRT monitor), which in turn was replaced by a pattern mask (####) for 16.6 ms. Then, the target stimulus was presented until the participant responded or 2.1 sec had elapsed. All the stimuli were presented in the same spatial location. Participants were instructed to press a “sí” [yes] button when the letter string formed a Spanish word and to press a “no” button when the letter string did not form a word. The instructions stressed both speed and accuracy. The experimental phase (288 trials; i.e., 144 word trials and 144 nonword trials) was preceded by sixteen practice trials. There were two self-paced breaks during the experimental phase. Participants did not mention having perceived any of the primes when asked after the experiment. The entire session lasted 12–16 minutes.

## Results

Incorrect responses (8.1% for word targets and 9.6% for nonword targets) and lexical decision times less than 200 or greater than 2000 ms (0.8% for word targets and 1.6% for nonword targets) were excluded from the latency analyses. Note that a different outlier exclusion criterion (e.g., excluding those response times beyond 2.5 standard deviations from the participants’ mean) would have produced a very similar pattern of data to the one reported here. In addition, three words (ruin, gula, musa) were removed from the analyses because of their elevated error rate (above 35%). The mean lexical decision times and the error rate per condition are shown in Table 1. As in the Perea et al. [8, 9] and Vergara-Martínez et al. [10] experiments, we focused on the specific research questions rather than conducting an unfocused omnibus Analysis of Variance (ANOVA). In particular, to examine whether there are differences between matched-case vs. mismatched-case identity primes, we conducted ANOVAs on the mean response time and error data with a 2 (prime-target relationship: matched-case identity, mismatched-case identity) x 2 lexicality (word, nonword) x 2 (type of stimulus: cross-case visually similar [SIM letters] vs. cross-case visually dissimilar [DIS letters]) design. For comparison purposes with previous experiments, we also conducted an ANOVA to examine the repetition priming effect on the latency data for words and nonwords (i.e., unrelated condition minus mismatched-case identity condition). All the statistical analyses were conducted over subjects (*F*1) and over items (*F*2).

**Table 1:** Mean Lexical Decision Times (RTs, in Milliseconds) and Percentages of Errors (ERs) in each Condition for Word and Nonword Targets in the Experiment.

	Words				Nonwords			
	Similar letters		Dissimilar letters		Similar letters		Dissimilar letters	
	RT	ER	RT	ER	RT	ER	RT	ER
Matched-case ID	638	9.6	623	6.4	714	8.8	728	9.4
Mismatched case ID	655	7.4	637	7.1	744	10.0	738	8.3
Unrelated	684	9.8	664	8.2	743	11.4	740	9.4
Mismatched-matched ID	17	-2.2	14	0.7	30	1.2	10	-1.1
Repetition priming	29	2.4	27	1.1	-1	1.4	2	1.1

### Matched-case vs. mismatched-case identity primes

The ANOVA on the latency data showed that responses to word targets were, on average, 84 ms faster than the responses to nonword targets, *F*1(1,26) = 52.82, *η*^*2*^ = .67, *p* < .001; *F*2(1,92) = 27.72, *η*^*2*^ = .23, *p* < .001. More importantly, responses to the target stimuli were, on average, 18 ms slower when preceded by a mismatched-case identity prime than when preceded by a matched-case identity prime, *F*1(1,26) = 12.51, *η*^*2*^ = .33, *p* = .002; *F*2(1,92) = 11.07, *η*^*2*^ = .11, *p* = .001. Importantly, this mismatched-case disadvantage for identity primes was similar for word and nonword targets (16 ms for word targets and 20 ms for nonword targets), as deduced from the lack of interaction between lexicality and prime-target relationship (both *F*s < 1). Furthermore, this mismatched-case disadvantage for identity primes occurred to a similar degree for SIM and DIS stimuli, as deduced from the lack of interaction between type of stimulus and prime-target relationship, both *p*s > .13. The interaction between type of stimulus and lexicality approached significance in the analyses by participants, *F*1(1,26) = 3.28, *p* = .082; *F*2 < 1—this reflected a small overall advantage of SIM vs. DIS stimuli for words, but not for nonwords. The other effects/interactions did not approach significance, all *F*s < 1.

The only significant effect in the ANOVA on the error data was the lexicality x prime-target relationship x type of stimulus interaction, *F*1(1,26) = 6.80, *η*^*2*^ = .19, *p* = .021; *F*2(1,92) = 11.07, *η*^*2*^ = .11, *p* = .001—none of the simple effects tests comparing the matched- vs. mismatched identity conditions approached significance (all *p*s > .10).

### Repetition identity priming

The ANOVA on the latency data showed the usual pattern of masked identity priming effects (see [17] for evidence of masked repetition priming with deaf readers): 1) a significant masked repetition priming effect for words (28 ms; *F*1(1,26) = 21.07, *η*^*2*^ = .45, *p* < .001; *F*2(1,46) = 9.41, *η*^*2*^ = .18, *p* = .004) that was similar in magnitude for SIM and DIS words (29 vs. 27 ms, respectively); and 2) a null masked priming effect for nonwords (1 ms; both *F*s < 1).

## Discussion

The main findings of the present masked priming lexical decision experiment with deaf readers can be summarized as follows. Similarly to hearing readers, nominal identity does not override physical identity in nonwords (GEDA-GEDA faster than geda-GEDA). But the crucial finding of the experiment is that, unlike hearing readers, nominal identity does not override physical identity in words either (REAL-REAL faster than real-REAL). Finally, cross-case visual similarity of primes/targets does not play an important role in modulating the magnitude of masked priming effects: 1) the differences between matched-case vs. mismatched-case identity pairs were similar for SIM and DIS words (see [8– 10] for similar evidence with hearing readers); and 2) the magnitude of masked identity priming was similar for SIM and DIS words (see [8, 9, 26] for similar evidence with hearing readers).

As indicated in the Introduction, the word/nonword dissociation that occurs with matched-case vs. mismatched-case identity primes (i.e., REAL-REAL similar to real-REAL, but GEDA-GEDA faster than geda-GEDA) rules out the proposal that the mismatched retinotopic representations of letters are mapped prelexically onto orthographic and phonological representations in a purely bottom-up fashion (see [10] for discussion). Instead, this word/nonword dissociation can be better explained by top-down influences from phonological-lexical codes that help override physical differences for word stimuli—this mechanism would not be operative for nonwords because they do not have stored representations. Here we have shown that, under the special scenario of impoverished phonological coding (presumably non-operative during the early stages of visual word processing, as deduced from the lack of masked phonological priming in deaf readers compared to hearing readers: [15, 17]; see [30–32]), this dissociation disappears: an advantage of matched-case over mismatched-case identity primes was obtained for both nonwords and words. This favors the view that the lack of differences between matched-case vs. mismatched-case identity priming for words occurs because of top-down feedback from phonological-lexical information taking place very early during prime coding. What we should note here is that the matched-case advantage for identity primes with deaf readers is not due to a scale effect (i.e., one might argue that the matched-case advantage may originate from slow response times). Indeed, response times in the Perea et al. [9] experiment with developing (Grade 5) readers were substantially slower than those obtained here—using a comparable set of word/nonword pairs—and there were no signs of a matched-case identity advantage for word pairs. We also conducted a post hoc analysis to examine whether the matched-case identity advantage for words was modulated by age. To that end, we created two age groups (younger than 30 [N = 13] vs. older than 30 [N = 14]). Results showed that the matched-case identity advantage for words was similar in the two groups (14 vs. 18 ms, respectively). Furthermore, as Perea et al. [8] showed, the lack of a matched-case advantage occurs not only for high-frequency words, but also for low-frequency words (around 3.6–10 occurrences per million). We also conducted a post hoc analysis to examine the relationship between the participants’ oral skills (in a 0–10 scale) and the matched-case identity advantage for words. The idea is that oral skills could be serving as a proxy for phonological coding (i.e., the assumption is that the better the oral skills of the deaf reader, the better the phonological coding abilities). The Pearson correlation was not close to significance, *r* = .19, *p* = .33. While we acknowledge that one should be cautious of the results of post hoc analyses, this null effect is consistent with the claim that “the use of phonological codes in reading is not a determinant of reading skills in the deaf population” (p. 2248; [19]). Finally, a question for additional research is whether very low frequency or irregular words (where grapheme-to-phoneme rules do not apply consistently) benefit from top-down feedback from phonological-lexical codes when comparing matched-case vs. mismatched-case identity pairs in hearing readers.

However, it may be difficult to determine whether the observed effects are the result of only phonological information (or lack thereof), because orthography and phonology are confounded in alphabetic orthographies. Therefore, it is important to examine the viability of other accounts of the present data. A potential explanation is that the mismatched-case disadvantage that occurs for words in deaf readers is driven by factors other than phonology. In particular, the differences between REAL-REAL and real-REAL in deaf readers could be the result of reduced top-down feedback in general in deaf individuals who might rely more heavily on visual information. While this explanation would accommodate the disadvantage of real-REAL over REAL-REAL in the response times for words, it runs into difficulties because, as occurs with hearing readers, visual similarity across prime and target does not seem to modulate the differences between matched-case and mismatched-case identity pairs. Had deaf participants relied on visual information, the mismatched-case identity disadvantage would have been larger for DIS than for SIM words. However, the mismatched-case disadvantage was virtually the same for the two types of words (17 and 14 ms, respectively). Furthermore, the magnitude of masked identity priming effects relative to the unrelated priming condition was also similar for SIM and DIS words (29 and 27 ms, respectively; see also [8, 9, 26] for converging evidence with hearing readers). This common pattern across hearing readers and deaf readers reveals that, even in a situation where the phonological codes may not be fully available in the early stages of word processing, the cognitive system is still able to build abstract representations. Indeed, Polk et al. [23] implemented a neural Hebbian network in which abstract letter representations of cross-case visually dissimilar letters (e.g., “a” and “A”) were created on the basis of visually similar surrounding contexts in the absence of phonology (e.g., as in CAP and cap; note that c/C and p/P are visually similar across case). Nonetheless, what we should note here is that, for nonwords, the matched-case vs. mismatched-case difference was numerically larger for SIM than for DIS stimuli in deaf readers (30 vs 10 ms, respectively)—the critical interaction was not significant, though. Further research using on-line measures (e.g., ERP waves; see [33]) would be necessary to examine in detail the similarities/differences in the time course of processing between SIM and DIS pairs with hearing and deaf readers. Finally, although the lack of a hearing group as a control could be considered a weakness of the present study, it is important to stress that the similar response times or ERP waves (N250, N400) for pairs like REAL-REAL and real-REAL is a solid finding that has been reported with hearing adults and developing readers in different languages.

In sum, previous research has shown that, regardless of their reading skill, deaf readers’ phonological encoding is poor or absent at the very early stages of visual word processing, as deduced from the null masked phonological priming effects in this population [15, 17]. The data from the present experiment are consistent with the idea that, unlike their hearing counterparts, deaf readers do not benefit from top-down processing from phonological-lexical information that help override the physical differences between prime and target in the early stages of word processing. This pattern suggests that word recognition in deaf readers is “qualitatively different” from word recognition in hearing readers, as Bélanger et al. [15] had claimed. More research is necessary to fully understand the similarities/dissimilarities in visual word processing between hearing and deaf readers.

## Appendix

### List of words and nonwords in the experiment

SIM words: kiwi; casi; seis; vino; cine; vivo; cien; mito; cita; suma; misa; cima; nuca; mina; cuna; test; unir; iris; musa; imán; timo; mimo; ruin; bici

(Approximate English translations: kiwi; almost; six; wine; cinema; alive; hundred; myth; date; sum; mass; top; nape; mine; cradle; test; bind; iris; muse; magnet; swindle; mime; ruin; bike)

DIS words: beca; gran; edad; real; alta; vale; dedo; raro; dama; reír; leve; amar; paga; rama; rata; leña; aula; rana; baúl; vara; atar; cala; gula; laca

(Approximate English translations: scholarship; great; age; real; high; voucher; finger; rare; lady; laugh; slight; love; pay; branch; rat; firewood; classroom; frog; trunk; rod; tie; creek; gluttony; hairspray)

SIM nonwords: muwi; bitu; cuek; cevi; cuve; vume; nais; misi; tise; conu; cise; nuvo; vuca; cika; vitu; rint; etuc; ivir; vimu; itén; nunu; kicu; voit; nuta

DIS nonwords: bame; gleb; emab; gial; ebta; dake; gete; deru; heda; daed; dage; ameg; gada; dodi; lida; geda; aete; wede; meer; gobe; etad; cehe; beñe; reva
